# Health-related quality of life of adolescents living with HIV treated at the HIV Clinic at the National Institute of Pediatrics at Mexico City

**DOI:** 10.1192/j.eurpsy.2022.1123

**Published:** 2022-09-01

**Authors:** D.B. Montoya Moya, D. Molina, J. Ordoñez Ortega

**Affiliations:** 1 Instituto Nacional de Pediatria, Salud Mental, Ciudad de Mexico, Mexico; 2 INSTITUTO NACIONAL DE PEDIATRIA, Child And Adolescence Psychiatry, MEXICO, Mexico; 3 Instituto Nacional de Pediatria, Infectology Department, Ciudad de Mexico, Mexico

**Keywords:** health-related quality of life, HIV, Quality of Life

## Abstract

**Introduction:**

ATR for children has successfully increase survival to adolescence. Health-related quality of life (HRQoL) is relevant to evaluate the impact of the disease on well-being in adolescents living with HIV (ALH). Kidscreen-52 questionnaire is validated in mexican adolescents to measure HRQoL

**Objectives:**

To evaluate health related quality of life in a sample of 22 mexican ALH

**Methods:**

A sample of ALH in treatment at the HIV Clinic during 2021, were evaluated with Kidscreen-52 by a child psychiatrist. Statistics included non parametric tests and Cohen “d” and “r” size effect to compare T means between ALH and Kidscreen-52 standardized scores.

**Results:**

Mean age:14.4+2.5. Gender: 11(50%)boys, 11(50%)girls. ALH showed significantly lower scores in all domains. Girls reported lower scores in physical well-being(p=0.047) and autonomy (p=0.023). Orphan ALH had lower scores in mood and emotions (p=0.021)

**Table:** 

KIDSCREEN-52	ALH MEAN/SD	KIDSCREEN-52 MEAN/SD	COHEN’S”d”	“r”
PYSICAL WELL-BEING	18.45+3.9	42.6+6.6	- 4.4	-0. 91
PSYCHOLOGICAL WELL-BEING	23.04+6.03	51.2+8.7	- 3.7	-0. 88
MOOD	25.3+6.13	44.8+7.5	- 2.8	-0. 81
SELF-PERCEPTION	20.27+3.22	47.3+7.6	- 4.6	-0. 91
AUTONOMY	18.09+4.9	46.6+9.4	- 3.8	-0. 88
SOCIAL SUPPORT AND PEERS	24+5.1	51.0+9.4	-3.5	-0. 87
PARENTS AND HOME LIFE	20.95+6.2	48.6+9.4	- 3.4	- 0.86
FINANCIAL RESOURCES	9.04+3.21	44.7+7.3	- 6.3	-0. 95
SCHOOL ENVIROMENT	19.73+7.13	53.3+7.9	- 4.46	-0. 91
SOCIAL ACCEPTANCE	12.91+2.11	46.3+9.6	- 4.8	- 0.92

**Conclusions:**

- HRQoL were significantly lower in ALH. -Girls showed significantly lower scores in physical well-being and autonomy. - ALH orphans showed significantly lower scores in mood and emotions domain

**Disclosure:**

No significant relationships.

